# Synthesis, Microtubule-Binding Affinity, and Antiproliferative Activity of New Epothilone Analogs and of an EGFR-Targeted Epothilone-Peptide Conjugate

**DOI:** 10.3390/ijms20051113

**Published:** 2019-03-05

**Authors:** Fabienne Zdenka Gaugaz, Andrea Chicca, Mariano Redondo-Horcajo, Isabel Barasoain, J. Fernando Díaz, Karl-Heinz Altmann

**Affiliations:** 1 ETH Zürich, Department of Chemistry and Applied Biosciences, Institute of Pharmaceutical Sciences, 8093 Zürich, Switzerland; fabienne.gaugaz@gmx.net; 2 Institute of Biochemistry and Molecular Medicine, University of Bern, 3012 Bern, Switzerland; andrea.chicca@ibmm.unibe.ch; 3 Centro de Investigaciones Biológicas, Consejo Superior de Investigaciones Científicas, 28040 Madrid, Spain; horcajo@cib.csic.es (M.R.-H.); i.barasoain@cib.csic.es (I.B.); fer@cib.csic.es (J.F.D.)

**Keywords:** cancer, drug discovery, epothilone, medicinal chemistry, microtubule-stabilizing agents, prodrug, total synthesis, tumor-targeting

## Abstract

A new simplified, epoxide-free epothilone analog was prepared incorporating an N-(2-hydroxyethyl)-benzimidazole side chain, which binds to microtubules with high affinity and inhibits cancer cell growth in vitro with nM potency. Building on this scaffold, a disulfide-linked conjugate with the purported EGFR-binding (EGFR, epidermal growth factor receptor) peptide GE11 was then prepared. The conjugate retained significant microtubule-binding affinity, in spite of the size of the peptide attached to the benzimidazole side chain. The antiproliferative activity of the conjugate was significantly lower than for the parent scaffold and, surprisingly, was independent of the EGFR expression status of cells. Our data indicate that the disulfide-based conjugation with the GE11 peptide is not a viable approach for effective tumor-targeting of highly potent epothilones and probably not for other cytotoxics.

## 1. Introduction

Tubulin modulators represent an important class of anticancer drugs that includes microtubule-stabilizing agents (MSA) such as paclitaxel, docetaxel, and ixabepilone as well as microtubule disrupters such as vinblastine and vincristine [1]. However, in spite of their clinical relevance and therapeutic success, drugs acting on the tubulin/microtubule system are still associated with significant side effects, due to the ubiquitous nature of their target protein tubulin [2]. Thus, the specific targeting of tubulin modulators, and cytotoxic agents in general, to tumors, has emerged as an important strategy in anticancer drug discovery and development. The most advanced approach towards the tumor-targeting of cytotoxic agents is their attachment to tumor-specific antibodies, with three antibody-drug conjugates (ADCs) being currently approved for cancer treatment in humans [3,4]; for two of these ADCs, the cytotoxic payload is a microtubule disrupter, i.e., monomethyl auristatin for brentuximab vedotin and the maytansine derivative DM-1 for trastuzumab emtansine. Alternative targeting moieties are peptides [5] or small molecules that interact specifically with proteins on tumor cell surfaces (e.g., folic acid [6] or synthetic organic molecules [7,8]).

When designing tumor-targeted conjugates of cytotoxic agents, two of the most crucial questions relate to the site of the attachment of the linker between the bioactive parent molecule and the targeting moiety and the nature of the linker moiety itself. Numerous linker modalities have been investigated in the literature, including protease/peptidase-cleavable linkers or simple disulfide groups (often in combination with a self-immolative structural element) [9]. Interestingly, the approved ADC trastuzumab emtansine incorporates a non-cleavable linker and the release of the active drug moiety is based on lysosomal degradation of the targeting antibody, leaving behind a construct composed of DM-1, the connector between DM-1 and the antibody, and the lysine reside to which the connector is attached as the pharmacologically active agent. We have recently shown for the first time that this construct does indeed bind to tubulin with similar affinity as the parent compound DM-1 (as its methylsulfanyl derivative) [10]. 

**Figure 1:**
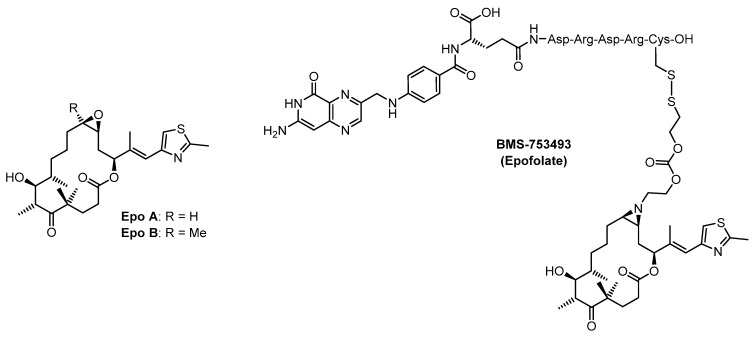
Molecular structures of epothilones A and B and of the tumor-targeted epothilone conjugate BMS-753493 (epofolate).

Epothilones A and B (Epo A and B) (Figure 1) are 16-membered myxobacterial macrolides that exhibit potent microtubule-stabilizing activity [11,12]. Both compounds exert strong antiproliferative effects on human cancer cells in vitro [12,13] and in human xenograft models in mice, including cell lines and tumors with taxol resistance [14,15,16]. The cytotoxic activity of Epo A is similar to that of taxol, whereas Epo B is generally more potent than taxol by at least one order of magnitude, with sub-nanomolar IC_50_ values for most cell lines [12,13].

Epothilones have been the subject of extensive SAR studies and at least eight epothilone-type agents have been advanced to clinical trials in humans in cancer [17,18], including the FDA-approved anticancer drug ixabepilone (the lactam analog of Epo B) [19]; in addition, 12,13-deoxy-Epo B (Epo D) has been investigated in Phase I clinical trials for Alzheimer’s disease [20]. While development of most of these compounds has been terminated (including the development of Epo D for Alzheimer’s disease), an analog termed utidelone (or UTD1) is currently undergoing Phase III clinical studies for breast cancer treatment in combination with capecitabine [21]. (Very surprisingly (and, in fact, irritatingly), several publications on this compound have appeared in the peer-reviewed literature, without its structure being revealed in any of these papers) [22]. Known attempts at the development of tumor-targeted derivatives of epothilones so far have been limited to the highly elaborate folate conjugate BMS-753493 (epofolate) (Figure 1) [23], where linker cleavage involves intracellular disulfide reduction followed by intramolecular ester cleavage to produce an N-(2-hydroxyethyl) derivative of aziridine-Epo A as the cytotoxic effector molecule.

**Figure 2:**
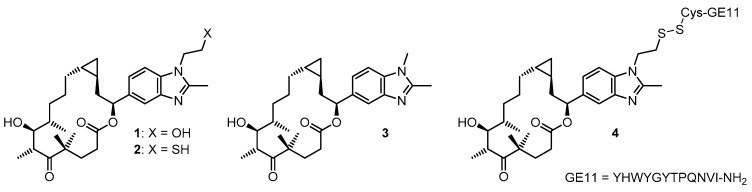
Molecular structures of epothilone analogs **1**–**3** and of the tumor-targeted epothilone peptide conjugate **4**.

In this paper, we report a different approach towards the design of epothilone-based tumor-targeted conjugates that is based on the benzimidazole-containing, structurally simplified cyclopropyl-*trans*-epothilone A analog **1** (Figure 2). We have previously shown that the related epothilone analog **3** (Figure 2) shows similar antiproliferative activity as Epo B [24]; at the same time, the presence of the larger 2-hydroxyethyl substituent on the benzimidazole moiety was found to be well tolerated for a corresponding cyclopropyl-Epo B derivative [25]. Lastly, the replacement of the epoxide moiety in natural epothilones by a cyclopropane ring is expected to lead to enhanced metabolic stability.

In the actual tumor-targeted conjugate **4**, the thiol analog of **1** (i.e., **2**) is connected to the side chain of a Cys residue added to the N-terminus of the epidermal growth factor receptor (EGFR)-binding peptide GE11 [26]. Reductive disulfide cleavage of this conjugate, thus, would directly lead to the cytotoxic effector molecule without the need for a subsequent immolative step (as in BMS-753493). 

The EGFR (HER1, ErbB1) is a 170 kDa transmembrane glycoprotein with an intracellular tyrosine kinase effector domain [27]. Enhanced EGFR signaling is associated with increased proliferation, differentiation, angiogenesis, invasion and metastasis and overexpression or mutation of EGFR is characteristic of a variety of human tumors [28]. Importantly in the context of this work, the EGFR is the targeted cell surface element for one of the marketed ADCs (trastuzumab emtansine). GE11 is a 12-amino acid residue peptide of the sequence YHWYGYTPQNVI, which has been reported to bind to EGFR with high affinity and specificity (K_d_ 22 nM), to be internalized preferentially into cells with high EGFR expression levels and to accumulate in EGFR-overexpressing tumor xenografts after i.v. administration in vivo [26]. GE11 has been investigated as a tumor-targeting device, e.g., in liposomes [29], nanoparticles [30], or polymeric prodrugs [31], but also for receptor imaging purposes [32]. 

## 2. Results and Discussion

### 2.1. Chemistry

**Scheme 1:**
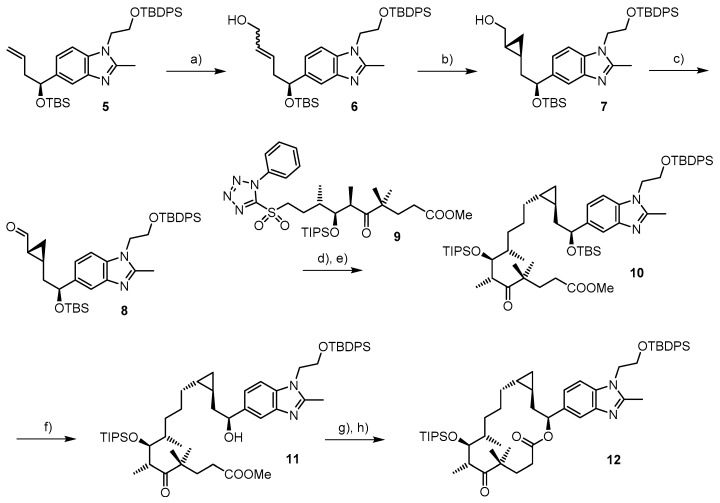
(**a**) *cis*-But-2-ene-1,4-diol, Hoveyda-Grubbs II (20 mol %), DCM, rt, 22 h, 80%, *E*/*Z* ca. 3:1. (**b**) i. Et_2_Zn, CH_2_I_2_, DCM, −10 °C, 15 min; ii. **6**, (4*S,5S*)-2-butyl-*N,N,N’,N’*-tetramethyl-1,3,2-dioxaborolane-4,5-dicarboxamide, DCM, 0 °C to rt, 2h, 78%. (**c**) DMP, DCM, rt, 5 h, 92%. (**d**) LiHMDS, THF, −78 °C to rt, 2 h, 72%. (**e**) NBSH, TEA, DCM, rt, 16 h, 98%. (**f**) CSA, DCM/MeOH 1:1, rt, 16 h, quant. (**g**) LiOH, *t*-BuOH/H_2_O 4:1, rt, 2.5 h, 98%. (**h**) 2,4,6 trichlorobenzoyl chloride, DMAP, TEA, toluene, rt, 2 h, 51%. Abbreviations: DCM: dichloromethane; DMP: Dess–Martin periodinane; CSA: camphorsulfonic acid; DMAP: 4-dimethylaminopyridine; LiHMDS: lithium hexamethyldisilazane; NBSH: *o*-nitrobenzenesulfonylhydrazide; TEA: triethylamine; THF: tetrahydrofuran.

The synthesis of both epothilone analog **1** as well as GE11-conjuagte **4** proceeded through the protected macrolactone **12** as a key precursor; as outlined in Scheme 1, the latter was obtained from the known olefin **5** [25] as an advanced intermediate. Cross metathesis of **5** (83% ee) with *cis*-buten-1,4-diol gave allylic alcohol **6** as an inseparable ca. 3:1 mixture of *E*/*Z* isomers, which was submitted as such to Charette cyclopropanation [33]. The cyclopropanation reaction furnished a mixture of products **7** that could not be separated. According to ^1^H-NMR, two major isomers were present in a ca. 9:1 ratio, but it was not determined if these isomers were distinguished by the configuration of the hydroxyl-bearing stereocenter (as a consequence of the imperfect stereochemical purity of olefin **5**) or by the stereochemistry of the cyclopropane moiety. Oxidation of **7** with Dess–Martin periodinane furnished aldehyde **8** as a mixture of three detectable isomers in a 1:0.14:0.03 ratio (based on the aldehyde signal in the ^1^H-NMR spectrum), which again proved to be inseparable. Aldehyde **8** was obtained in a 58% overall yield for the three-step sequence from olefin **5**.

The elaboration of aldehyde **8** into the epothilone macrocyclic framework in a first step entailed Julia–Kocienski olefination with sulfone **9** (Scheme 1) [34]. The reaction was best carried out under Barbier conditions in the presence of two equivalents of LiHMDS, which furnished the desired olefin in a 72% yield with ca. 2/1 selectivity (based on ^1^H-NMR). The low selectivity of the olefination reaction was inconsequential, as the double bond was reduced in the next step with diimide to provide the fully protected *seco* ester **10** in quantitative yield. Either 2,4,6-triisopropylbenzenesulfonylhydrazide (TPSH) [35] or *o*-nitrobenzenesulfonylhydrazide (NBSH, as a cheaper alternative) [36] could be employed as a diimide source, with both methods delivering **10** in excellent yields. Selective cleavage of the benzylic silyl-ether with CSA in DCM/MeOH 1:1 then furnished alcohol **11** in quantitative yield. (Careful inspection of the NMR spectra of **10** and **11** suggested the presence of at least one minor isomer in both cases, whose content was difficult to quantify, however). Saponification of methyl ester **11** gave a crude acid that was directly submitted to Yamaguchi macrolactonization [37], to give the fully protected macrolactone **12** in 51% yield after purification by preparative RP-HPLC. Ca. 500 mg of this key intermediate were prepared in a purity that was sufficient for subsequent manipulations, thus highlighting the practicability of the process developed. 

**Scheme 2:**
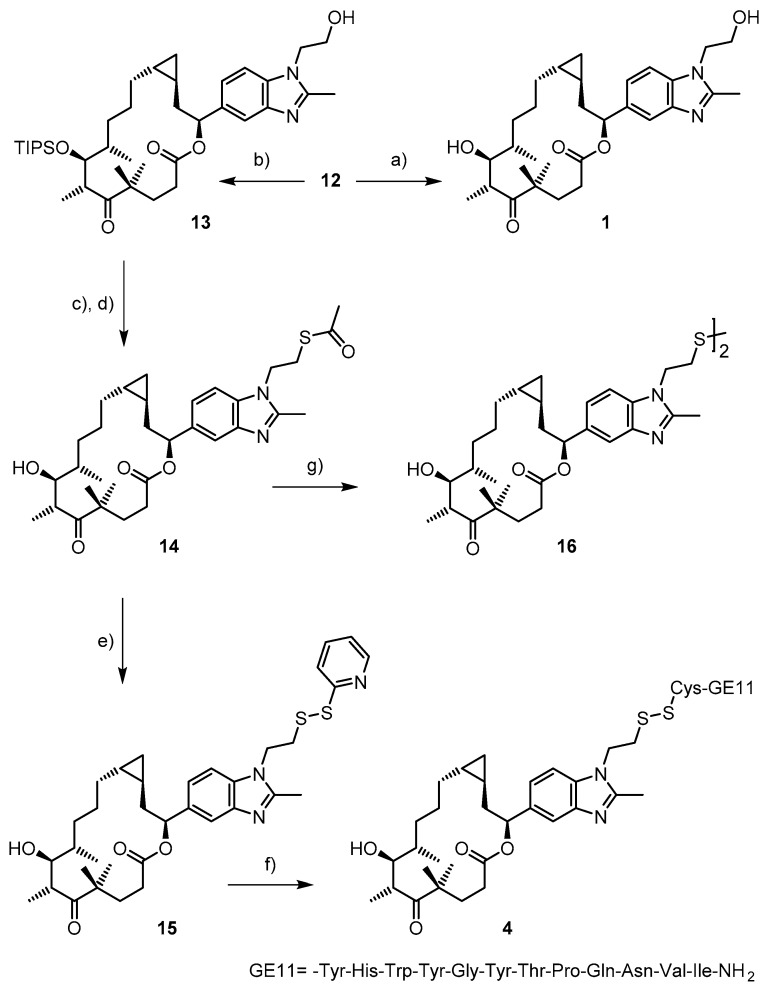
(**a**) HF∙py, MeCN, rt, 16 h, 33% after HPLC. (**b**) TASF, DMF, 0 °C, 2 h, quant. (**c**) PPh_3_, DEAD, AcSH, Et_2_O, 0 °C to rt, 3.5 h. (**d**) HF∙py, pyridine, MeCN, rt, 53 h, 87% (2 steps). (**e**) 2,2′-dipyridyl disulfide, K_2_CO_3_, MeOH, rt, 30 min, 79%. (**f**) CysGE11, MeOH, rt, 2 h, 15% after HPLC. (**g**) K_2_CO_3_, MeOH, rt, 20 min, 24% after RP-HPLC. Abbreviations: TASF: tris(dimethylamino)sulfonium difluoromethylsilicate; DMF: dimethyl formamide; DEAD: diethyl azodicarboxylate.

As illustrated in Scheme 2, global deprotection of **12** with HF∙pyridine gave *trans*-cyclopropyl epothilone **1** in 33% yield after purification by preparative RP-HPLC. Alternatively, **12** could be selectively deprotected at the primary hydroxyl group with TASF (tris(dimethylamino)sulfonium difluoromethylsilicate) [38] to give the partially protected macrolactone **13** in quantitative yield. Mitsunobu reaction with thioacetic acid as the nucleophile then gave a thioester derivative from which the O-TIPS (triisopropylsilylether) protecting group was removed with HF∙pyridine, to provide thioacetate **14**. The latter could be directly converted into the activated mixed disulfide **15** by reaction with 2,2′-dipyridyl disulfide under slightly basic conditions. Reaction of **15** with CysGE11 then provided the desired epothilone-GE11 conjugate **4**. 

While the thioester moiety of **14** could be readily cleaved with K_2_CO_3_/MeOH, the resulting free thiol **2** could not be isolated as a pure material. Disulfide formation was already observed between elution of the material from the HPLC column and lyophilization of the sample. Only disulfide **16** could be isolated and characterized.

### 2.2. Biological Assessment

In order to provide a rational framework for the biological assessment of the epothilone peptide conjugate **4**, EGFR was quantified in cell lines that were to be used for those experiments, i.e., A431 epidermoid squamous cell carcinoma cells, SW480 colorectal adenocarcinoma cells, and HEK293 embryonic kidney cells. A431 cells have been reported to highly overexpress EGFR [39]; consistent overexpression of EGFR has also been described for SW480 cells [40]. In contrast, HEK293 cells were previously found not to express EGFR [39]. Expression levels of EGFR were assessed by the treatment of cells with cetuximab followed by FACS-based quantification of cetuximab with an Alexa Fluor 647 goat anti-human antibody. In line with the existing literature, EGFR expression levels were found to be highest in A431 cells, followed by SW480 cells. HEK293 cells gave fluorescence intensities at the level of the isotype control (Figure S1). Therefore, **4** would have been expected to display strongest cytotoxicity against A431 and SW480 cells, while HEK293 cells should have been largely insensitive to the action of the conjugate. As will be discussed below, the experimental results did not conform to these predictions. 

The stability of the epothilone-GE11 conjugate **4** in complete cell culture medium was evaluated under conditions resembling those encountered in the cytotoxicity assays. Thus, an 8.6 μM solution of **4** in RPMI with 10% FBS was incubated for 3 days at 37 °C and subsequently analyzed by RP-HPLC. Only minor degradation of **4** (<10%) was observed under these conditions (data not shown).

**Figure 3:**
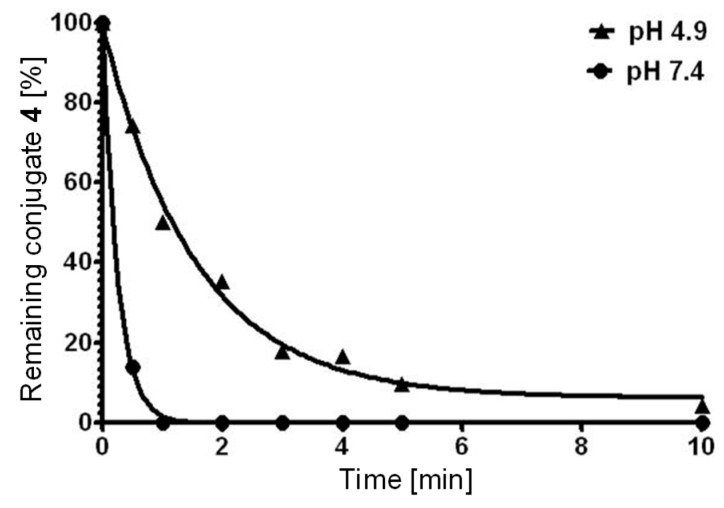
Kinetics of glutathione (GSH)-mediated reduction of epothilone-peptide conjugate **4** (173 µM **4**, 10 mM GSH, pH 4.9 (endosome-like conditions), triangles, or pH 7.4 (cytoplasm-like conditions), circles). Conjugate **4** was added to the respective GSH solutions as a solution in phosphate buffer pH 7.4 at room temperature, to reach a total concentration of 173 μM. Samples were quenched at different time points with 10% metaphosphoric acid and analyzed by RP-HPLC. The peak areas for **4** in the RP-HPLC chromatograms were normalized against the peak area of a 200 µM solution of **4** in the corresponding GSH-free buffers.

The reductive cleavage of conjugate **4** was analyzed under conditions mimicking those found in the endosome and the cytoplasm of cancer cells [41]. Endosome-like conditions were assumed to be reproduced by a 10 mM solution of glutathione (GSH) in acetate and phosphate buffer pH 4.9; cytoplasm-like conditions entailed 10 mM GSH in phosphate buffer pH 7.4. Under both conditions, rapid disappearance of **4** was observed with apparent first order kinetics (Figure 3).

The half-life of **4** was 1.1 min under endosome-like conditions, while no residual conjugate was detectable even 1 min after addition to the glutathione solution under cytoplasm-like conditions (estimated half-life of ca. 0.2 min). LC/MS analysis of cleavage solutions showed that the products obtained at both pH values included the mono- and dimeric CysGE11 peptide, thiol-containing epothilone analog **2**, the mixed disulfide of **2** with glutathione, and dimer **16**. The mixed disulfide of **2** with glutathione appeared very early in the reaction (Figure S2); it was later transformed into **2** and **16**. The shorter half-life of **4** under cytoplasm-like conditions is in line with the fact that disulfide reduction is pH-dependent and has been shown to be significantly slower at lower pH [41].

The binding affinity of epothilone analogs **1**, **14**, and **16** and of targeted conjugate **4** for microtubules was determined as previously described by Díaz and co-workers, using partly cross-linked microtubules and determining the changes in fluorescence anisotropy upon displacement of the fluorescent taxol derivative Flutax-2 [42,43] (Figure S3). Not unexpectedly, the microtubule-binding affinity of the simplified epothilone analog **1** even exceeds that of natural Epo A (K_b_ of 7.85 × 10^7^ M^−1^ vs. 3.17 × 10^7^ M^−1^ for Epo A; Table 1); this observation is in line with existing SAR data for other epothilone analogs that are derived from *trans*-Epo A and/or incorporate a benzimidazole side chain [24,25,44], although analog **1** has not been investigated previously. Compared to **1**, the K_b_ for thioacetate **14** is considerably lower (>10-fold), but its microtubule-binding affinity is still comparable to that of other epothilone analogs that have shown nanomolar antiproliferative activity against different cancer cell lines [45]. Intriguingly, analog **16** and conjugate **4** exhibit similar microtubule-binding affinity as thioacetate **14**, in spite of the significant enlargement in size of the substituent group on the benzimidazole side chain. Importantly, the targeting peptide CysCE11 showed no relevant microtubule binding. 

The antiproliferative activity of the targeted epothilone conjugate **4** was assessed for A431, SW480, and HEK293 cells, in comparison with epothilone analogs **1** and **16**. Of the three compounds investigated, conjugate **4** proved to be the least potent by one order of magnitude (Table 2).

While the lower activity of **4** compared to **1** against A431 and SW480 cells could potentially be rationalized by a lower rate of receptor-mediated uptake vs. uptake by passive diffusion (which is likely to be operative for **1**) and/or the efficiency of formation of **2** from **4** in, and its release from, the endosomal compartment, the lack of selectivity between EGFR-expressing A431 and SW480 cells and essentially EGFR-free HEK293 cells is difficult to explain. This finding may point to cleavage of the disulfide bond in the extracellular space, and the passive diffusion of **2** into the cells (assuming that passive diffusion of conjugate **4** through the cell membrane followed by intracellular disulfide reduction is essentially excluded). Should this indeed be the case, the same mechanism may also underlie the activity of **4** against A431 and SW480 cells, without the involvement of EGFR-mediated endocytosis. In this context, it should be remembered that 10% disulfide cleavage or less would already account for the observed IC_50_ values, if one assumes the (elusive) epothilone **2** to exhibit similar activity as **1**. Extracellular disulfide cleavage as the source of the antiproliferative activity of **4** would thus still be compatible with the stability data obtained for the compound in cell culture medium (vide supra). Preliminary experiments aiming at the quantification of intracellular concentrations of **4**, **2**, or **16** did not produce any interpretable results.

In a broader context, we note that recycling endosomes, late endosomes, and lysosomes, in contrast to our own basic assumption, had been suggested to provide a reducing, rather than oxidizing environment, even prior to the start of our work on **4** [46]. At the same time, careful inspection of the literature suggests that, while the GE11 peptide does seem to allow selective targeting of EGFR-overexpressing cells by nanoparticles [30] or polymeric prodrugs [31], the selectivity that has been achieved is rather moderate. In line with these observations, the reported K_d_ of 22 nM of GE11 for the EGFR could not be reproduced in subsequent studies by other laboratories. Thus, Levitzki and co-workers determined the IC_50_ for the displacement of [^125^I]-EGF from the EGFR by GE11 to be >1 mM (!) (vs. 5.1 nM for EGF) [47]; more recently, Lin and co-workers determined the K_d_ of GE11 for the EGFR as 459 µM by means of surface plasmon resonance [48]. Similar findings have been reported by Sihver and co-workers in the context of studies on potential GE11-derived PET imaging agents [49]. Thus, in retrospect, neither the choice of the GE11 peptide as an EGFR-targeting moiety nor its combination with a disulfide linker may have been optimal. 

The antiproliferative activity of disulfide **16** is similar to that of analog **1**, in spite of its >10-fold lower microtubule-binding affinity. While microtubule-binding affinity is not necessarily linearly correlated with cell growth inhibition, this finding may point to thiol-containing epothilone analog **2** as the species mainly responsible for the antiproliferative effects of **16**. It is easily conceivable for disulfide **16** to readily cross the cellular membrane by passive diffusion (the molecular weight of **16** is 1054 vs., e.g., 856 for taxol, which easily enters cells); immediate reduction in the cytoplasmic compartment would then produce **2**. However, we do not have any experimental data at this point to support this (plausible) mechanistic hypothesis. 

## 3. Materials and Methods

### 3.1. Chemistry

All non-aqueous reactions were carried out under anhydrous conditions and under an argon atmosphere unless otherwise noted. CH_2_Cl_2_ was distilled from CaH; THF, Et_2_O, benzene, and toluene were distilled from Na/benzophenone. All other absolute solvents were purchased from Fluka (absolute over molecular sieves). Commercial chemicals were used without further purification, unless otherwise noted. 

Solvents for extractions, column chromatography, and thin layer chromatography (TLC) were purchased as commercial grade and distilled prior to use. TLC was performed on Merck TLC aluminum sheets (silica gel 60, F254). Spots were visualized with UV light (254 nm) or through staining with an aqueous solution of phosphomolybdic acid, cerium sulfate, and sulfuric acid (CPS). Chromatographic purification of products was performed by flash chromatography (FC) using Fluka silica gel 60 for preparative column chromatography (particle size 40–63 µm). Organic solutions were concentrated by rotary evaporation at 40 °C and approximately 20 mbar. The compounds were further dried under high vacuum (0.01–0.001 mbar). Yields refer to compounds isolated after FC, unless otherwise specified. NMR spectra were recorded on a Bruker AV-400 400 MHz or a Bruker AV-500 500 MHz NMR spectrometer (Bruker Biospin AG, Fällanden, Switzerland) at 298 K. NMR spectra are referenced relative to the residual hydrogen signal of the deuterated solvent (^1^H 7.26 ppm, ^13^C 77.0 ppm for CDCl_3_). All ^13^C spectra were measured with complete proton decoupling. For inseparable diastereomeric mixtures, only signals of the major diastereomer are reported. Spin multiplicities are reported as follows: s = singlet, d = doublet, t = triplet, q = quartet, quint = quintet, sext = sextet, m = multiplet, mc = centered multiplet, br = broad signal; J = coupling constant in Hz. Infrared spectra (IR) were recorded on a Jasco FT/IR-6200 instrument. Positions of absorption bands are given in wavenumbers [cm^−1^]. Optical rotations were measured on a Jasco P-1020 polarimeter and are reported as follows: ${\left[\alpha \right]}_{D}^{24}$: concentration (g/100 mL) and solvent. High resolution mass spectra (HRMS) were recorded by the ETH Zürich MS service on a Varian IonSpec Ultima (ESI) or a Waters Micromass Autospec Ultima spectrometer (EI). RP-HPLC was carried out on a Merck Hitachi device (column oven L-2350, diode array detector L-2450, autosampler L-2200, pump L-2130) using a Waters Symmetry C18 column, 3.5 µm, 4.6 × 100 mm, at a flow rate of 1 mL/min for analytical purposes; a Waters Symmetry C18 column, 5 µm, 7.8 × 100 mm, at a flow rate of 2 mL/min was employed for semi-preparative applications. The column temperature for analytical and semi-preparative applications was 40 °C. Preparative RP-HPLC was carried out on a Gilson device (Gilson (Schweiz) AG, Mettmenstetten, Switzerland) with a Waters Symmetry C18 column (Waters AG, Baden-Dättwil, Switzerland), 5 µm, 19 × 100 mm, at a flow rate of 25 mL/min at room temperature. Peaks were detected at 280, 254, or 220 nm. The Cys-GE11 peptide was custom-made by Biosyntan GmbH (Berlin, Germany).

**(*S,E*)-5-((*tert*-Butyldimethylsilyl)oxy)-5-(1-(2-((*tert*-butyldiphenylsilyl)oxy)ethyl)-2-methyl-1H-benzo[d]imidazol-5-yl)pent-2-en-1-ol (6)**

A solution of Hoveyda-Grubbs II catalyst (104.6 mg, 0.167 mmol) in DCM (0.2 mL) was added to a solution of olefin **5** (500 mg, 0.835 mmol) and *cis*-2-butene-1,4 diol (206 µL, 2.504 mmol) in DCM (6 mL) at rt. The solution was stirred for 22 h at rt. It was then concentrated in vacuo and purified by FC (EtOAc/MeOH 100:1) to yield 420.2 mg of **6** as a ca. 3/1 mixture of *E*/*Z* isomers. 

*R*_f_ = 0.31 (EtOAc/MeOH 20:1). ${\left[\alpha \right]}_{D}^{20}$ = −16.8 (c = 1.1 in CHCl_3_). ^1^H NMR (400 MHz, CDCl_3_): *δ* = 7.60 (1H, s), 7.24–7.43 (10H, m), 7.13 (1H, dd, *J* = 1.43, 8.30 Hz), 7.05 (1H, d, *J* = 8.30 Hz), 5.65 (2H, m), 4.79 (1H, dd, *J* = 5.41, 6.95 Hz), 4.22 (2H, t, *J* = 5.59 Hz), 4.04 (2H, d, *J* = 4.68 Hz), 3.90 (2H, t, *J* = 5.59 Hz), 2.54 (3H, s), 2.37-2.65 (2H, m), 0.93 (9H, s), 0.88 (9H, s), 0.02 (3H, s), −0.14 ppm (3H, s). ^13^C NMR (100 MHz, CDCl_3_): *δ* = 152.30, 142.38, 139.24, 135.51, 134.53, 132.63, 131.64, 129.95, 129.72, 127.90, 120.25, 116.37, 108.97, 75.43, 63.77, 62.10, 45.88, 44.51, 26.74, 26.00, 19.02, 18.39, 14.09, −4.40, −4.76. IR: *ν* = 3245, 2953, 2929, 2857, 2357, 1520, 1471, 1429, 1404, 1360, 1254, 1110, 1085, 1007, 940 835, 776, 739, 702 cm^−1^. HRMS (ESI): *m/z* calcd for C_37_H_52_N_2_O_3_Si_2_ + H^+^: 629.3589 [*M* + H^+^]; found 629.3589.

**((1*R*,2*S*)-2-((*S*)-2-((*tert*-Butyldimethylsilyl)oxy)-2-(1-(2-((*tert*-butyldiphenylsilyl)oxy)ethyl)-2-methyl-1H-benzo[d]imidazol-5-yl)ethyl) cyclopropyl)methanol (7)**

Diiodomethane (0.16 mL, 2 mmol) was slowly added to a solution of diethyl zinc (1 M in hexane, 1 mL, 1 mmol) in DCM (1 mL) at −10 °C and the solution was stirred for 15 min. (Note the section about EXPLOSIONS in [33]). A preformed solution of allylic alcohol **6** (97 mg, 0.154 mmol) and (4*S*,5*S*)-2-butyl-*N*,*N*,*N*’,*N*’-tetramethyl-1,3,2-dioxaborolane-4,5-dicarboxamide (97 µL, 0.386 mmol) in DCM (3 mL) was added at 0 °C. The solution was stirred for 2 h at rt. It was then washed with 1 M HCl, and the aqueous phase was re-extracted with DCM. The organic layers were transferred to an Erlenmeyer flask, 2 M NaOH and H_2_O_2_, 30%, 6:1, were added in one portion, and the mixture was vigorously stirred for 5 min. The organic layer was then successively washed with 1 M HCl and sat. aq. Na_2_SO_3_, dried over MgSO_4_ and concentrated in vacuo. Purification of the residue by FC (EtOAc/MeOH 100:1) yielded 77.7 mg of cyclopropane **7** as a mixture of at least two isomers (see text). 

*R*_f_ = 0.39 (EtOAc/MeOH 95:5). ${\left[\alpha \right]}_{D}^{20}$ = −21.1 (c = 1.1 in CHCl_3_). ^1^H NMR (400 MHz, CDCl_3_): *δ* = 7.63 (1H, s), 7.24–7.44 (10H, m), 7.14 (1H, dd, *J* = 1.39, 8.28 Hz), 7.05 (1H, d, *J* = 8.28 Hz), 4.84 (1H, t, *J* = 6.17 Hz), 4.22 (2H, t, *J* = 5.68 Hz), 3.89 (2H, t, *J* = 5.68 Hz), 3.37 (2H, m), 2.54 (3H, s), 1.51–1.81 (2H, m), 0.93 (9H, s),0.89 (9H, s), 0.79 (1H, m), 0.65 (1H, m), 0.30 (2H, m), 0.03 (3H, s), −0.12, (3H, s). ^13^C NMR (100 MHz, CDCl_3_): *δ* = 152.28, 142.41, 139.64, 135.53, 134.49, 132.66, 129.96, 127.91, 120.31, 116.41, 108.94, 75.49, 67.13, 62.13, 45.90, 45.56, 26.75, 26.05, 21.52, 19.04, 18.39, 14.10, 13.92, 9.81, −4.40, −4.78 ppm; IR: *ν* = 3266, 2953, 2929, 2857, 1520, 1471, 1429, 1403, 1360, 1253, 1111, 1086, 939, 836, 776, 739, 703 cm^−1^. HRMS (ESI): *m/z* calcd for C_38_H_54_N_2_O_3_Si_2_ + H^+^: 643.3746 [*M* + H^+^]; found 643.3744.

**(1R,2S)-2-((S)-2-((tert-Butyldimethylsilyl)oxy)-2-(1-(2-((tert-butyldiphenylsilyl)oxy)ethyl)-2-methyl-1H-benzo[d]imidazol-5-yl)ethyl) cyclopropanecarbaldehyde (8)**

DMP (19.8 mg, 0.047 mmol) in DCM (1 mL) was slowly added to a solution of alcohol **7** (30 mg, 0.047 mmol) in DCM (1 mL) and the solution was stirred for 5 h at rt. More DMP (10 mg, 0.024 mmol) in DCM (1 mL) was added at this point and stirring was continued for 1 h. The reaction mixture was evaporated and the crude residue was directly purified by FC (EtOAc/MeOH 100:1), to yiel 27.4 mg aldehyde **8** (92%) as a mixture of 3 isomers in a ratio of 1: 0.14: 0.03.

*R*_f_ = 0.73 (EtOAc/MeOH 95:5). ${\left[\alpha \right]}_{D}^{20}$ = −28.5 (c = 0.4 in CHCl_3_). ^1^H NMR (400 MHz, CDCl_3_): *δ* = 8.87 (1H, d, *J* = 5.57 Hz), 7.61 (1H, s), 7.25–7.43 (10H, m), 7.11 (1H, dd, *J* = 1.31, 8.30 Hz), 7.06 (1H, d, *J* = 8.30 Hz), 4.85 (1H, dd, *J* = 5.02, 6.88 Hz), 4.23 (2H, t, *J* = 5.68 Hz), 3.89 (2H, t, *J* = 5.68 Hz), 2.54 (3H, s), 1.93 (1H, m), 1.56 (2H, m), 1.49 (1H, m), 1.20 (1H, m), 0.93 (9H, s), 0.89 (9H, s), 0.85 (1H, m), 0.03 (3H, s), −0.15 ppm (3H, s). ^13^C NMR (100 MHz, CDCl_3_): *δ* = 201.03, 152.42, 142.31, 139.09, 135.53, 134.64, 132.65, 129.99, 127.93, 120.33, 116.18, 109.11, 75.10, 62.14, 45.94, 44.51, 30.54, 26.76, 26.02, 19.39, 19.05, 18.33, 14.42, 14.05, −4.42, −4.82. IR: *ν* = 2953, 2928, 2856, 2352, 1708, 1521, 1471, 1463, 1429, 1402, 1361, 1255, 1111, 1086, 937, 836, 778, 745, 736, 703 cm^−1^. HRMS (ESI): *m/z* calcd for C_39_H_52_N_2_O_3_Si_2_ + H^+^: 641.3589 [*M* + H^+^]; found 641.3592.

**(6*R*,7*S*,8*S*,*E*)-Methyl-11-((1*R*,2*S*)-2-((*S*)-2-((*tert*-butyldimethylsilyl)oxy)-2-(1-(2-((*tert*-butyldiphenyl-silyl)oxy)ethyl)-2-methyl-1H-benzo[d]imidazol-5-yl)ethyl)cyclopropyl)-4,4,6,8-tetramethyl-5-oxo-7-((triisopropylsilyl)oxy) undec-10-enoate (10A)**

A solution of LiHMDS (618 mg, 3.969 mmol) in THF (1 mL) was added to a solution of sulfone **9** [34] (1.07 g, 1.680 mmol) and aldehyde **8** (1.3 g, 2.028 mmol) in THF (5 mL) at −78°C. The mixture was stirred for 1 h at −78 °C and allowed to warm to rt over a period of 1 h. After quenching the reaction with sat. aq. NH_4_Cl, the solution was extracted with EtOAc, and the combined extracts were dried over MgSO_4_ and concentrated in vacuo. Purification of the residue by FC (Hex/EtOAc/MeOH 1:1:0.01) yielded 1.4 g of olefin **10A** as a mixture of isomers (see text). 

*R*_f_ = 0.88 (EtOAc/MeOH 20:1). ${\left[\alpha \right]}_{D}^{20}$ = −13.3 (c = 0.8 in CHCl_3_). ^1^H NMR (400 MHz, CDCl_3_): *δ* = 7.60 (1H, s), 7.24–7.42 (10H, m), 7.10 (1H, m), 7.04 (1H, m), 5.32 (1H, m), 4.95 (1H, m), 4.79 (1H, m), 4.22 (2H, t, *J* = 5.30 Hz), 4.04 (1H, m), 3.89 (2H, t, *J* = 5.39 Hz), 3.65 (3H, s), 3.15 (1H, m), 2.54 (3H, s), 2.22 (2H, m), 1.88-2.06 (2H, m), 1.83 (2H, m,), 1.74 (1H, m), 1.47 (1H, m), 1.28 (1H, m), 1.19 (3H, s), 1.10 (28H, m), 0.93 (12H, br s), 0.88 (9H, s), 0.82-0.86 (1H, m), 0.41 (1H, t, *J* = 6.66 Hz), 0.03 (3H, s), −0.15 (3H, s). ^13^C NMR (100 MHz, CDCl_3_): *δ* = 217.98, 174.14, 152.20, 142.55, 140.00, 135.54, 135.06, 134.48, 132.67, 129.96, 127.92, 126.34, 120.43, 116.35, 108.77, 77.32, 75.66, 62.14, 51.77, 47.70, 46.28, 45.91, 43.42, 40.23, 35.12, 34.44, 29.89, 26.76, 26.07, 24.80, 21.56, 19.04, 18.61, 18.59, 18.38, 17.83, 16.68, 16.45, 15.97, 14.15, 13.66, −4.38, −4.76. IR: *ν* = 2926, 2856, 2362, 1741, 1697, 1521, 1462, 1430, 1389, 1360, 1254, 1112, 1087, 883, 836, 702 cm^−1^; HRMS (ESI): *m/z* calcd for C_62_H_98_N_2_O_6_Si_3_ + H^+^: 1051.6805 [*M* + H^+^]; found 1051.6796.

**(6*R*,7*S*,8*S*)-Methyl11-((1R,2*S*)-2-((*S*)-2-((*tert*-butyldimethylsilyl)oxy)-2-(1-(2-((*tert*-butyl-diphenylsilyl)oxy)ethyl)-2-methyl-1H-benzo[d]imidazol-5-yl)ethyl)cyclopropyl)-4,4,6,8-tetra-methyl-5-oxo-7-((triisopropylsilyl) oxy) undecanoate (10)**

Triethylamine (TEA) (103 µL, 0.742 mmol) was slowly added to a solution of olefin **10A** (39 mg, 0.037 mmol) and NBSH (161 mg, 0.742 mmol) in DCM (2 mL) at rt, and the reaction mixture was stirred overnight. As the reaction was not complete at this point, additional TEA (200 µL, 1.439 mmol) was added over a period of 4 h. After an additional 20 h, more NBSH (50 mg, 0.23 mmol) and TEA (100 µL, 0.72 mmol) were added, and stirring was continued for 16 h. The solution was then washed with brine, dried over MgSO_4_, and concentrated in vacuo. Purification of the residue by FC (Hex/EtOAc 2:1) yielded 38.1 mg of saturated ester **10** (98%) as a mixture of isomers (see text). 

*R*_f_ = 0.81 (EtOAc/Hex 1:1). ${\left[\alpha \right]}_{D}^{20}$ = −20.6 (c = 1.0 in CHCl_3_). ^1^H NMR (400 MHz, CDCl_3_): *δ* = 7.60 (1H, s), 7.23–7.43 (10H, m), 7.12 (1H, dd, *J* = 1.30, 8.35 Hz), 7.03 (1H, d, *J* = 8.35 Hz,), 4.77 (1H, dd, *J* = 5.48, 7.32 Hz), 4.22 (2H, t, *J* = 5.59 Hz), 4.03 (1H, dd, *J* = 2.58, 6.10 Hz), 3.89 (2H, t, *J* = 5.59 Hz), 3.64 (3H, s), 3.11 (1H, q, *J* = 6.70 Hz), 2.54 (3H, s), 2.22 (2H, m), 1.84 (3H, m), 1.49 (1H, m), 1.21-1.45 (5H, m), 1.19 (3H, s), 1.10 (29H, m), 0.94 (3H, m), 0.93 (9H, s), 0.88 (9H, s), 0.49 (1H, m), 0.41 (1H, m), 0.13 (2H, m), 0.03 (3H, s), −0.14 (3H, s). ^13^C NMR (100 MHz, CDCl_3_): *δ* = 217.94, 174.11, 152.16, 142.53, 140.11, 135.53, 134.45, 132.67, 129.94, 127.91, 120.39, 116.46, 108.78, 77.04, 75.96, 62.13, 51.73, 47.63, 46.71, 45.89, 42.78, 40.31, 34.82, 34.55, 32.35, 29.87, 27.91, 26.75, 26.07, 24.88, 24.80, 19.08, 19.03, 18.58, 18.55, 18.40, 15.93, 15.88, 15.78, 14.15, 13.53, 11.85, −4.36, −4.76. IR: *ν* = 2929, 2861, 2357, 1740, 1697, 1521, 1471, 1429, 1390, 1361, 1254, 1111, 1086, 1006, 883, 836, 776, 740, 703, 505 cm^−1^. HRMS (ESI): *m/z* calcd for C_62_H_100_N_2_O_6_Si_3_ + H^+^: 1053.6962 [*M* + H^+^]; found 1053.6957.

**(6*R*,7*S*,8*S*)-Methyl11-((1*R*,2*S*)-2-((*S*)-2-(1-(2-((*tert*-butyldiphenylsilyl)oxy)ethyl)-2-methyl-1H-benzo[d]imidazol-5-yl)-2-hydroxyethyl)cyclopropyl)-4,4,6,8-tetramethyl-5-oxo-7-((triisopropyl-silyl)oxy)undecanoate (11)**

Camphorsulfonic acid (CSA) (0.3 g, 1.291 mmol) was added to a solution of silyl ether **10** (0.38 g, 0.361 mmol) in DCM/MeOH 1:1 (16 mL). The solution was stirred overnight at rt and then neutralized with sat. aq. NaHCO_3_. The aqueous layer was extracted with DCM, and the combined organic layers were dried over MgSO_4_ and concentrated in vacuo. Purification of the residue by FC (Hex/EtOAc 9:1) yielded 0.34 g of partially protected seco acid **11** (quant) as a mixture of isomers (see text). 

*R*_f_ = 0.67 (EtOAc/Hex 9:1). ${\left[\alpha \right]}_{D}^{20}$ = −14.7 (c = 1.1 in CHCl_3_). ^1^H NMR (400 MHz, CDCl_3_): *δ* = 7.65 (1H, s), 7.24–7.42 (10H, m), 7.17 (1H, dd, *J* = 1.41, 8.34 Hz), 7.04 (1H, d, *J* = 8.34 Hz), 4.84 (1H, t, *J* = 6.55 Hz), 4.22 (2H, t, *J* = 5.66 Hz), 4.03 (1H, dd, *J* = 2.53, 6.10 Hz,),3.89 (2H, t, *J* = 5.66 Hz), 3.64 (3H, s), 3.12 (1H, q, *J* = 6.62 Hz), 2.55 (3H, s), 2.22 (2H, m), 1.83 (3H, m), 1.61 (1H, m), 1.48 (1H, m), 1.28 (3H, m), 1.19 (3H, s), 1.09 (30H, m), 0.94 (9H, s,), 0.92 (3H, m), 0.48 (2H, m), 0.17 ppm (2H, m). ^13^C NMR (100 MHz, CDCl_3_): *δ* = 217.96, 174.14, 152.57, 142.74, 138.92, 135.53, 134.80, 132.65, 129.98, 127.91, 120.31, 116.60, 109.25, 77.05, 75.53, 62.02, 51.76, 47.62, 45.88, 44.26, 42.81, 40.27, 34.74, 34.54, 32.34, 29.86, 27.94, 26.77, 24.88, 24.79, 19.06, 19.00, 18.58, 18.55, 15.92, 15.76, 15.64, 14.13, 13.54, 11.51. IR: *ν* = 3229, 2930, 2865, 2356, 1739, 1696, 1521, 1462, 1429, 1403, 1390, 1111, 1087, 1007, 883, 740, 703, 504 cm^−1^. HRMS (ESI): *m/z* calcd for C_56_H_86_N_2_O_6_Si_2_ + H^+^: 939.6097 [*M* + H^+^]; found 939.6093.

**(6*R*,7*S*,8*S*)-11-((1*R*,2*S*)-2-((*S*)-2-(1-(2-((*tert*-Butyldiphenylsilyl)oxy)ethyl)-2-methyl-1H-benzo[d]imidazol-5-yl)-2-hydroxyethyl)cyclopropyl)-4,4,6,8-tetramethyl-5-oxo-7((triisopropylsilyl)oxy)undecanoic acid (11A)**

Lithium hydroxide monohydrate (223.1 mg, 5.555 mmol) was added to a solution of methyl ester **11** (1.04 g, 1.111 mmol) in *t*-BuOH/H_2_O 4:1 (30 mL). The solution was stirred for 2.5 h at rt. Water and 1 M HCI (to pH 5) were then added, and the solution was extracted with DCM. The combined organic extracts were dried over MgSO_4_ and evaporated in vacuo to furnish obtain 1.000 g of crude acid **11A** (98%) as a white powder. This material was used in the next step without further purification. *R*_f_ = 0.15 (EtOAc/MeOH 95:5). ${\left[\alpha \right]}_{D}^{20}$ = +16.4 (c = 0.7 in CHCl_3_). ^1^H NMR (400 MHz, CDCl_3_): *δ* = 7.71 (1H, s), 7.25–7.43 (10H, m), 7.35 (1H, m), 7.14 (1H, d, *J* = 8.46 Hz), 4.85 (1H, dd, *J* = 4.43, 10.00 Hz), 4.22 (2H, m), 4.05 (1H, dd, *J* = 2.98, 4.59 Hz), 3.89 (2H, t, *J* = 5.60 Hz), 3.18 (1H, m), 2.56 (3H, s), 2.21–2.36 (2H, m), 2.07 (1H, m), 1.93 (2H, m), 1.67 (1H, m), 1.50 (3H, m), 1.31 (3H, s), 1.25 (1H, m), 1.24 (1H, m), 1.06–1.15 (29H, m), 0.95 (3H, m), 0.94 (9H, s), 0.53 (1H, m), 0.10–0.23 (2H, m), −0.04 (1H, m). ^13^C NMR (100 MHz, CDCl_3_): *δ* = 219.00, 176.43, 152.33, 139.77, 139.20, 135.51, 134.03, 132.53, 130.09, 127.98, 119.90, 117.33, 110.31, 75.76, 75.75, 61.93, 47.58, 45.88, 44.04, 41.65, 40.94, 35.32, 34.25, 33.60, 30.46, 27.96, 26.78, 24.81, 24.36, 19.08, 18.92, 18.52, 18.47, 16.05, 15.00, 13.34, 13.22, 13.13, 11.50. IR: *ν* = 2960, 2930, 2865, 2358, 2338, 1698, 1520, 1472, 1428, 1407, 1390, 1362, 1111, 1088, 883, 773, 741, 703 cm^−1^. HRMS (ESI): *m/z* calcd for C_55_H_84_N_2_O_6_Si_2_ + H^+^: 925.5941 [*M* + H^+^]; found 925.5937.

**(1*S*,3*S*,10*R*,11*S*,12*S*,16*R*)-3-(1-(2-((*tert*-Butyldiphenylsilyl)oxy)ethyl)-2-methyl-1H-benzo[d]imidazol-5-yl)-8,8,10,12-tetramethyl-11-((triisopropylsilyl)oxy)-4-oxabicyclo[14.1.0] heptadecane-5,9-dione (12)**

A solution of acid **11A** (988 mg, 1.067 mmol) in toluene (20 mL) was slowly added to a solution of TEA (297 µL, 1.135 mmol), 2,4,6 trichlorobenzoyl chloride (184 µL, 1.174 mmol) and DMAP (170 mg, 1.388 mmol) in toluene (500 mL) at rt. The mixture was stirred for 2 h and the reaction was then quenched with sat. aq. NaHCO_3_. The solution was extracted with EtOAc, and the combined extracts were dried over MgSO_4_ and then concentrated in vacuo. Purification of the residue by FC (Hex/EtOAc/MeOH 1:1:0.01) followed by preparative RP-HPLC gave 493 mg of slightly impure macrolactone **12** (51%). Preparative RP-HPLC: H_2_O with 0.1% TFA (A)/CH_3_CN (B). 10% B to 80% over 20 min, retention time: 8.54 min.

*R*_f_ = 0.35 (EtOAc: Hex 1:1). ${\left[\alpha \right]}_{D}^{20}$ = −15.7 (c = 1.0 in CHCl_3_). ^1^H NMR (400 MHz, CDCl_3_): δ = 7.82 (s, 1H), 7.35 (m, 6H), 7.25 (m, 6H), 5.96 (d, *J* = 10.1 Hz, 1H), 4.34 (d, *J* = 5.2 Hz, 2H), 4.19 (dd, *J* = 7.3, 2.2 Hz, 1H), 4.00 (t, *J* = 4.5 Hz, 2H), 3.24 (p, *J* = 7.0 Hz, 1H), 2.79 (s, 3H), 2.35 (tdd, *J* = 27.4, 16.2, 5.2 Hz, 2H), 2.10 (dd, *J* = 14.5, 3.2 Hz, 1H), 1.95 (pd, *J* = 13.8, 5.3 Hz, 2H), 1.57–1.41 (m, 4H), 1.39 (s, 3H), 1.37–1.30 (m, 2H), 1.25 (s, 4H), 1.17 (d, *J* = 7.0 Hz, 4H), 1.12 (s, 18H), 1.06 (s, 4H), 0.98 (d, *J* = 6.8 Hz, 3H), 0.92 (s, 9H), 0.79–0.70 (m, 1H), 0.57 (dt, *J* = 12.9, 4.4 Hz, 1H), 0.22 ppm (tt, *J* = 9.4, 4.7 Hz, 2H). ^13^C NMR (101 MHz, CDCl_3_): δ = 218.62, 172.29, 151.22, 141.20, 135.31, 131.80, 131.56, 131.32, 130.44, 128.11, 124.67, 112.70, 111.30, 77.48, 76.79, 61.11, 47.35, 46.98, 43.58, 41.96, 39.70, 34.89, 34.47, 30.93, 26.79, 26.51, 25.15, 23.98, 18.99, 18.62, 18.58, 18.04, 17.02, 16.75, 16.00, 13.58, 11.80. IR: *ν* = 2932, 2865, 2359, 2338, 1731, 1695, 1521, 1463, 1429, 1390, 1255, 1111, 1088, 999, 981, 939, 883, 823, 754, 703, 677, 504 cm^−1^. HRMS (ESI): *m/z* calcd for C_55_H_82_N_2_O_5_Si_2_ + H^+^: 907.5835 [*M* + H^+^]; found 907.5837.

**(1*S*,3*S*,10*R*,11*S*,12*S*,16*R*)-11-Hydroxy-3-(1-(2-hydroxyethyl)-2-methyl-1H-benzo[d]imidazol-5-yl)-8,8,10,12-tetramethyl-4-oxabicyclo[14.1.0] heptadecane-5,9-dione (1)**

Fully protected macrolactone **12** (40 mg, 0.044 mmol) was dissolved in THF (3 mL) and HF∙pyridine (2 mL) was added. The solution was stirred overnight and the reaction then slowly quenched with aq. sat. NaHCO_3_. The solution was extracted with EtOAc and the combined organic phases were washed with aq. sat. NaHCO_3_, dried over MgSO_4_ and concentrated in vacuo. Purification of the residue by FC (CHCl_3_/MeOH/H_2_O/AcOH 85:13:1.5:0.5) was followed by preparative RP-HPLC purification. RP-HPLC: Injection of a solution of **1** in MeOH and elution with 0.1% TFA in H_2_O (A)/MeCN (B); 30% B for 2 min, then gradient to 60% B over 6 min, and then gradient to 90% B over 3 min. Preparative runs: 30% B for 2 min and then to 90% B over 10 min; retention time 4.67 min (prep). An amount of 9.11 mg of pure **1** was isolated (33%) with further mixed fractions.

*R*_f_ = 0.37 (CHCl_3_/MeOH/H_2_O/AcOH 85:13:1.5:0.5). ${\left[\alpha \right]}_{D}^{20}$ = −15.1 (c = 0.5 in MeOH). ^1^H NMR (500 MHz, MeOD): *δ* = 7.79 (d, *J* = 8.7 Hz, 1H), 7.69 (s, 1H), 7.54 (dd, *J* = 8.6, 0.7 Hz, 1H), 6.05 (dd, *J* = 11.0, 1.0 Hz, 1H), 4.53 (t, *J* = 5.1 Hz, 2H), 3.97–3.93 (m, 2H), 3.70 (dd, *J* = 9.9, 1.4 Hz, 1H), 3.29 (d, *J* = 6.7 Hz, 1H), 2.87 (s, 3H), 2.42–2.32 (m, 1H), 2.24 (dd, *J* = 8.6, 6.9 Hz, 1H), 2.21–2.12 (m, 1H), 2.03–1.94 (m, 1H), 1.87 (td, *J* = 14.4, 5.2 Hz, 1H), 1.71 (dd, *J* = 12.8, 5.2 Hz, 1H), 1.57 (d, *J* = 9.4 Hz, 1H), 1.48–1.42 (m, 2H), 1.35–1.24 (m, 3H), 1.20 (d, *J* = 6.7 Hz, 3H), 1.09 (dd, *J* = 16.0, 6.9 Hz, 1H), 1.02 (s, 3H), 0.99 (d, *J* = 6.6 Hz, 3H), 0.91–0.86 (m, 1H), 0.67–0.61 (m, 1H), 0.27–0.20 (m, 2H). ^13^C NMR (100 MHz, CDCl_3_): δ = 219.48, 172.13, 152.26, 140.88, 131.37, 131.17, 124.47, 112.29, 111.41, 76.71, 75.83, 59.56, 47.82, 47.55, 42.31, 41.63, 36.04, 34.37, 32.02, 30.94, 29.53, 25.54, 25.35, 22.78, 22.39, 18.62, 17.23, 16.98, 14.96, 12.03. IR: *ν* = 3414, 2966, 2934, 2878, 2363, 1729, 1674, 1460, 1426, 1200, 1175, 1138, 975, 798, 722 cm^−1^; HRMS (ESI): *m/z* calcd for C_30_H_44_N_2_O_5_ + H^+^: 513.3323 [*M* + H^+^]; found 513.3323.

**(1*S*,3*S*,10*R*,11*S*,12*S*,16*R*)-3-(1-(2-Hydroxyethyl)-2-methyl-1H-benzo[d]imidazol-5-yl)-8,8,10,12-tetramethyl-11-((triisopropylsilyl)oxy)-4-oxabicyclo[14.1.0] heptadecane-5,9-dione (13)**

A solution of dry TASF (28.2 mg, 0.102 mmol) in DMF (90 µL) was added to a solution of dry **12** (93 mg, 0.102 mmol, HPLC purified) in DMF (5 mL) at 0 °C, and the solution was stirred for 2 h. Phosphate buffer pH 7 was then added, and the solution was extracted with DCM, dried over MgSO_4_ and concentrated in vacuo. Purification of the residue by FC (EtOAc/MeOH 100:1) yielded 70 mg (quant) of alcohol **13** as a colorless oil.

*R*_f_ = 0.35 (EtOAc/MeOH 95:5). ${\left[\alpha \right]}_{D}^{20}$ = −21.3 (c = 0.7 in CHCl_3_). ^1^H NMR (400 MHz, CDCl_3_): *δ* = 7.51 (1H, s), 7.21 (1H, d, *J* = 8.22 Hz), 7.13 (1H, dd, *J* = 1.39, 8.33 Hz), 5.98 (1H, d, *J* = 10.20 Hz), 4.21 (2H, t, *J* = 5.17 Hz), 4.16 (1H, dd, *J* = 2.14, 7.10 Hz), 3.98 (2H, t, *J* = 5.17 Hz), 3.23 (1H, q, *J* = 7.00 Hz), 2.47 (3H, s), 2.82 (2H, m), 2.07 (1H, m), 1.92 (2H, m), 1.48 (3H, m), 1.35 (3H, s), 1.26 (5H, m), 1.17 (3H, d, *J* = 7.04 Hz), 1.11 (21H, s), 1.04 (3H, s), 0.98 (3H, d, *J* = 6.84 Hz), 0.71 (1H, m), 0.58 (1H, m), 0.20 (2H, m). ^13^C NMR (100 MHz, CDCl_3_): *δ* = 218.28, 172.26, 153.05, 142.16, 136.05, 134.68, 120.90, 115.93, 109.27, 77.65, 77.38, 60.78, 47.43, 46.54, 43.52, 42.17, 39.69, 34.86, 34.50, 31.06, 26.59, 25.49, 23.83, 18.68, 18.68, 18.61, 18.05, 17.27, 16.71, 16.06, 13.91, 13.57, 11.41. IR: *ν* = 3197, 2962, 2942, 2866, 2356, 2338, 1731, 1696, 1520, 1465, 1406, 1389, 1254, 1122, 1067, 1041, 999, 982, 883, 756, 677cm^−1^; HRMS (ESI): *m/z* calcd for C_39_H_64_N_2_O_5_Si + H^+^: 669.4657 [*M* + H^+^]; found 669.4649.

***S*-(2-(5-((1*S*,3*S*,10*R*,11*S*,12*S*,16*R*)-11-Hydroxy-8,8,10,12-tetramethyl-5,9-dioxo-4-oxabicyclo[14.1.0] heptadecan-3-yl)-2-methyl-1H-benzo[d]imidazol-1-yl)ethyl) ethanethioate (14)**

DEAD (35 µL, 0.219 mmol) was added to a solution of PPh_3_ (57.6 mg, 0.219 mmol) in Et_2_O (4 mL) at 0 °C, and the mixture was stirred for 1 h at that temperature. Thioacetic acid (17 µL, 0.241) was then added followed by slow addition of a solution of **13** (70 mg, 0.104 mmol) in Et_2_O (5 mL). The cooling bath was removed, and the solution was stirred at rt for 2.5 h. It was then washed with brine, and the aqueous solution was back-extracted with EtOAc. The combined organic extracts were dried over MgSO_4_ and concentrated in vacuo. Purification of the residue by FC (EtOAc/MeOH 100:1) gave 130 mg of protected thioacetate contaminated with triphenylphosphine oxide; 5 mg of **13** were recovered. The protected thioacetate was dissolved in THF (5 mL) and a total of 5 mL of pyridine and 9 mL of HF py were added in portions over a period of 53 h. NaHCO_3_ was then carefully added to the mixture to reach pH 7–8, and the solution was extracted with EtOAc. The combined organic extracts were dried over MgSO_4_ and concentrated in vacuo. Purification of the residue by FC (CHCl_3_/MeOH/H_2_O/AcOH 90:10:1:0.5 and CHCl_3_/MeOH/AcOH 95:4.5:0.5) yielded 52 mg (87% over 2 steps) of thioacetate **14** as a colorless oil. Ten milligrams of this material were purified by preparative RP-HPLC: Injection of a solution of **1** in MeOH and elution with 0.1% TFA in H_2_O (A)/CH_3_CN (B); 30% B for 2 min, then to 60% B over 6 min, and then to 90% B over 3 min. Preparative HPLC: 30% B for 2 min and then to 70% over 10 min. Retention time 6.60 (prep). An amount of 2.42 mg of pure **14** was recovered after HPLC (22% yield based on **13**).

*R*_f_ = 0.07 (CHCl_3_/MeOH/AcOH 95:4.5:0.5). ${\left[\alpha \right]}_{D}^{20}$ = −27.1 (c = 0.9 in CHCl_3_). ^1^H NMR (400 MHz, CDCl_3_): *δ* = 7.62 (1H, s), 7.36 (1H, d, *J* = 8.26 Hz), 7.20 (1H, dd, *J* = 1.39, 8.26 Hz), 6.00 (1H, dd, *J* = 1.62, 10.19Hz), 4.22 (2H, m), 3.78 (1H, dd, *J* = 3.09, 6.72 Hz), 3.29 (1H, q, *J* = 6.72 Hz), 3.16 (2H, m), 2.65 (3H, s), 2.38 (3H, s), 2.17–2.34 (2H, m), 2.11 (1H, m), 1.87–2.02 (2H, m), 1.54 (5H, m), 1.34 (3H, s), 1.25 (2H, m), 1.18 (3H, d, *J* = 6.79 Hz), 1.02 (3H, s), 1.00 (3H, d, *J* = 6.79 Hz), 0.89 (1H, m), 0.64 (1H, m), 0.56 (1H, m), 0.18 ppm (2H, m). ^13^C NMR (100 MHz, CDCl_3_): *δ* = 219.18, 195.28, 172.02, 152.20, 142.01, 136.43, 134.11, 121.33, 116.05, 109.1, 77.51, 76.07, 47.88, 43.16, 42.32, 41.93, 36.11, 34.44, 34.41, 31.11, 30.79, 29.86, 28.24, 25.69, 25.55, 22.26, 18.53, 17.33, 17.28, 15.21, 13.63, 11.01. IR: *ν* = 3267, 2962, 2930, 2856, 2363, 1725, 1691, 1521, 1458, 1402, 1252, 1133, 1039, 975, 754, 623 cm^−1^; HRMS (ESI): *m/z* calcd for C_32_H_46_N_2_O_5_S + H^+^: 571.3200 [*M* + H^+^]; found 571.3195.

**(1*S*,3*S*,10*R*,11*S*,12*S*,16*R*)-11-Hydroxy-8,8,10,12-tetramethyl-3-(2-methyl-1-(2-(pyridin-2-yldisulfanyl)ethyl)-1H-benzo[d]imidazol-5-yl)-4-oxabicyclo[14.1.0] heptadecane-5,9-dione (15)**

K_2_CO_3_ (5 mg) was added to a solution of **14** (11 mg, 0.019 mmol) and 2,2′-dipyridyl disulfide (5.1 mg, 0.023 mmol) in dry MeOH (2 mL), and the mixture was stirred at rt for 30 min. Brine was added and the solution was extracted with EtOAc. The combined organic extracts were dried over MgSO_4_ and concentrated. Purification of the residue by FC yielded 9.7 mg (79%) of activated disulfide **15**.

*R*_f_ = 0.76 (EtOAc/MeOH 9:1). ${\left[\alpha \right]}_{D}^{20}$ = −45.3 (c = 0.4 in CHCl_3_). ^1^H NMR (400 MHz, CDCl_3_): *δ* = 8.50 (1H, dq, *J* = 0.90, 4.02 Hz) 7.60 (3H, m), 7.22 (1H, d, *J* = 8.21 Hz), 7.14 (2H, m), 4.98 (1H, dd, *J* = 1.72, 10.18 Hz), 4.46 (2H, t, *J* = 7.31 Hz), 3.77 (1H, dd, *J* = 2.95, 6.66 Hz), 3.28 (1H, q, *J* = 6.76 Hz), 3.12 (2H, t, *J* = 7.33 Hz), 2.60 (3H, s), 2.13–2.38 (2H, m,), 2.07 (1H, m), 1.80–2.01 (2H, m), 1.54 (5H, m), 1.33 (3H, s), 1.27 (2H, m), 1.18 (3H, d, *J* = 6.76 Hz), 1.02 (3H, s), 1.00 (3H, d, *J* = 6.85 Hz), 0.87 (1H, m), 0.56–0.64 (2H, m), 0.18 (2H, m). ^13^C NMR (100 MHz, CDCl_3_): *δ* = 219.13, 171.99, 158.78, 152.24, 150.30, 142.73, 137.36, 136.23, 134.38, 121.56, 121.14, 120.68, 116.28, 108.83, 77.57, 76.07, 47.89, 42.94, 42.32, 42.01, 37.12, 36.13, 34.47, 34.43, 31.13, 29.90, 25.75, 25.59, 22.29, 18.56, 17.38, 17.28, 15.19, 14.26, 11.06. IR: *ν* = 3269, 2926, 2855, 2017, 1727, 1690, 1574, 1521, 1447, 1418, 1401, 1250, 975, 760 cm^−1^; HRMS (ESI): *m/z* calcd for C_35_H_47_N_3_O_4_S_2_ + H^+^: 638.3081 [*M* + H^+^]; found 638.3075.

**(*S*)-2-((*S*)-1-((6*R*,9*S*,12*S*,15*S*,18*S*,24*S*,27*S*)-12-((1H-imidazol-4-yl)methyl)-15-((1H-indol-3-yl)methyl)-6-amino-1-(5-((1*S*,3*S*,10*R*,11*S*,12*S*,16*R*)-11-hydroxy-8,8,10,12-tetramethyl-5,9-dioxo-4-oxabicyclo[14.1.0]heptadecan-3-yl)-2-methyl-1H-benzo[d]imidazol-1-yl)-9,18,24-tris(4-hydroxy-benzyl)-27-((*R*)-1-hydroxyethyl)-7,10,13,16,19,22,25-heptaoxo-3,4-dithia-8,11,14,17,20,23,26-heptaazaoctacosan-28-oyl)pyrrolidine-2-carboxamido)-N1-((S)-4-amino-1-(((S)-1-(((2S,3S)-1-amino-3-methyl-1-oxopentan-2-yl)amino)-3-methyl-1-oxobutan-2-yl)amino)-1,4-dioxobutan-2-yl)pentanediamide (4)**

**Figure 4:**
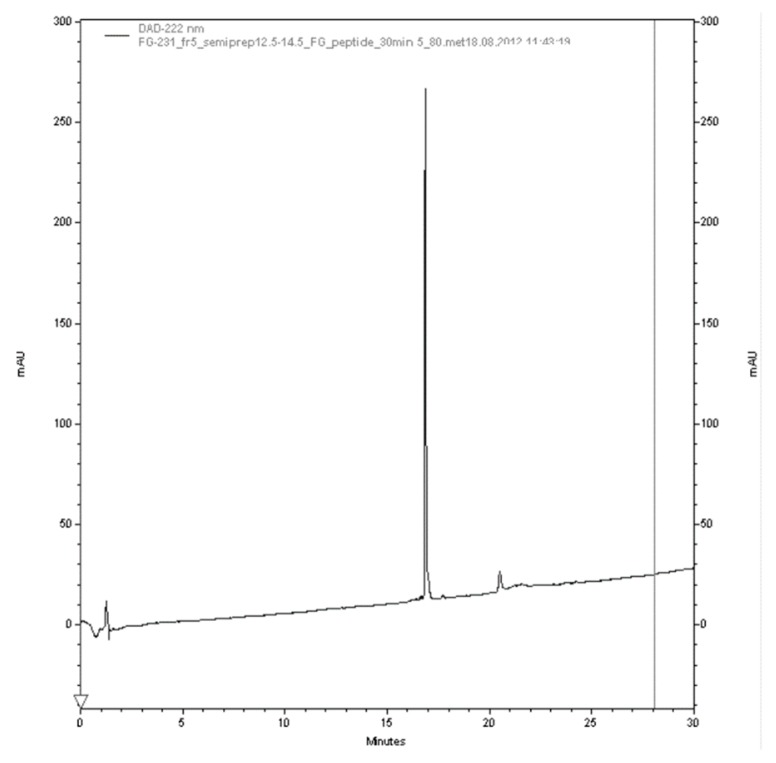
Analytical RP-HPLC trace of **4** after preparative RP-HPLC.

Cys-GE11 (11 mg, 0.0067 mmol) and **15** (4.3 mg, 0.0067 mmol) were dissolved in MeOH, and the solution was stirred at rt for 2 h. Evaporation of the solvent and purification of the residue by preparative HPLC yielded 2.45 mg (15%) of conjugate **4**. Analytical RP-HPLC (C18): H_2_O with 0.1% TFA (A)/H_2_O/CH_3_CN 8/2 with 0.05% TFA (B); gradient from 5% B to 80% B over 30 min. Preparative RP-HPLC: Gradient from 5% B to 70% B over 30 min. ${\left[\alpha \right]}_{D}^{20}$ = −1.5 (c = 0.2 in MeOH). IR: *ν* = 3853, 3821, 3751, 3735, 3710, 3690, 3676, 3670, 3649, 3629, 3587, 3567, 2361, 2334, 1684, 1559, 1540, 1507, 1457, 1208, 1186, 1138, 801, 725 cm^−1^; HRMS (ESI): *m/z* calcd for C_108_H_145_N_21_O_23_S_2_ + H^+^: 2169.0341 [*M* + H^+^]; found 2169.0355 (Figure 4).

**Dimer of (1*S*,3*S*,10*R*,11*S*,12*S*,16*R*)-11-hydroxy-3-(1-(2-mercaptoethyl)-2-methyl-1H-benzo[d] imidazol-5-yl)-8,8,10,12-tetramethyl-4-oxabicyclo[14.1.0]heptadecane-5,9-dione (16)**

K_2_CO_3_ (5 mg) was added to a solution of **14** (13 mg, 0.023 mmol) in dry MeOH (2 mL), and the mixture was stirred at rt for 20 min. Brine was added and the solution was extracted with EtOAc. The combined organic extracts were dried over MgSO_4_ and concentrated. The crude residue was purified by preparative HPLC. Although care was taken throughout the purification process to exclude air as much as possible, the material recovered after lyophilization contained disulfide **16** as the major component. Repurification of this material gave 3.51 mg (24%) of pure disulfide **16**. Preparative RP-HPLC: H_2_O with 0.1% TFA/CH_3_CN; 30% B for 2 min and then to 90% B over 10 min. Retention time 5.44 min/6.21 min for monomer/dimer (prep).

*R*_f_ = 0.76 (EtOAc/MeOH 9:1). ${\left[\alpha \right]}_{D}^{20}$ = −3.3 (c = 0.1 in MeOH). ^1^H NMR (500 MHz, MeOD): *δ* = 7.82 (d, *J* = 8.6 Hz, 2H), 7.70 (s, 2H), 7.57 (dd, *J* = 8.7, 1.3 Hz, 2H), 6.04 (d, *J* = 10.5 Hz, 2H), 4.73 (t, *J* = 6.5 Hz, 4H), 3.70 (dd, *J* = 9.7, 0.9 Hz, 2H), 3.26 (t, *J* = 6.3 Hz, 4H), 2.88 (s, 6H), 2.35 (ddd, *J* = 16.2, 12.9, 4.7 Hz, 2H), 2.22 (d, *J* = 14.5 Hz, 2H), 2.19–2.11 (m, 2H), 1.91 (dtd, *J* = 53.2, 13.6, 4.7 Hz, 5H), 1.75–1.63 (m, 2H), 1.60–1.51 (m, 3H), 1.48–1.41 (m, 4H), 1.40 (s, 6H), 1.30–1.23 (m, 3H), 1.19 (d, *J* = 6.7 Hz, 6H), 1.13–1.04 (m, 3H), 1.00 (s, 6H), 0.98 (d, *J* = 6.6 Hz, 6H), 0.92–0.82 (m, 2H), 0.63 (tt, *J* = 12.7, 6.4 Hz, 2H), 0.26–0.19 (m, 4H). ^13^C NMR (126 MHz, MeOD) *δ* = 218.32, 172.15, 152.13, 140.96, 131.64, 123.91, 119.94, 112.34, 111.38, 77.27, 77.16, 47.02, 43.94, 43.56, 41.44, 35.60, 35.13, 34.29, 34.01, 30.29, 28.02, 25.30, 24.68, 21.72, 18.12, 17.79, 17.22, 15.98, 10.82, 9.44. IR: *ν* = 2927, 2852, 2363, 2343, 2018, 1715, 1684, 1199, 1138, 974, 725 cm^−1^; HRMS (ESI): *m/z* calcd for C_60_H_86_N_4_O_8_S_2_ + H^+^: 1055.5960 [*M* + H^+^]; found 1055.5958.

### 3.2. Glutathione Cleavage Assay

For cytoplasm-like conditions, 100 µL of a 173 µM solution of conjugate **4** in degassed 100 mM phosphate buffer pH 7.4 were diluted with 400 µL of a degassed phosphate buffer and 500 µL of 20 mM glutathione in 100 mM phosphate buffer pH 7.4. Aliquots of 80 µL were removed at predefined time points, and the reaction was quenched with one volume of 10% metaphosphoric acid, giving a pH of 1 approximately. Fifty microliters of this solution were analyzed by analytical RP-HPLC. The same protocol was used for cleavage under endosome-like conditions, except that the glutathione solution was prepared in a 100 mM acetate buffer at pH 4.8, and the same buffer was also used to dilute the conjugate solution in a 100 mM phosphate buffer at pH 7.4. The final pH of the cleavage solution was 4.9.

Analytical RP-HPLC: H_2_O with 0.1% TFA (A)/acetonitrile/water 8/2 with 0.05% TFA (B). Linear gradient from 5% B to 80% B over 30 min. The percentage of remaining conjugate in the solutions was plotted against time (Figure 3). Analysis of the cleavage solutions by LC/MS provided information of the fractions of the various components in the redox mixture. As an example, Figure S2 shows the product distribution after 5 min of reaction time under endosome-like conditions.

### 3.3. Determination of Microtubule Binding Constants

Purified calf-brain tubulin and chemicals were obtained as previously described [50]. Stabilized, moderately crosslinked microtubules were prepared as reported in [42]. Binding constants of azathilones to stabilized microtubules were measured as previously described by Buey et al. [41]. For details of the experimental procedures and Flutax-2 displacement curves cf. Section SI.3 of the Supplementary Materials.

### 3.4. Antiproliferative Activity

Experiments were performed in RPMI (SW480 cells) or DMEM (HEK293 and A431 cells) medium (Gibco) supplemented with 2 mM glutamine, 10% fetal bovine serum, 100 IU/mL penicillin, 100 µg/mL streptomycin, and 1 µg/mL amphotericin B (all from Sigma) at 37 °C and 5% CO_2_. Cells were seeded into 96-well microtiter plates and compounds were added in DMSO in serial dilutions in triplicate. The final DMSO (dimethylsulfoxide) concentration was 0.1%. After 72 h, a WST-1 reagent [43] was added and the absorption was measured at 450 nm with a plate reader after 30 min to 4 h. The percentage of viable cells was calculated based on vehicle-treated cells and was plotted in GraphPadPrism. At least three independent experiments were performed.

A431 cells were obtained from CLS. The other cell lines were available in the laboratory. WST-1 was purchased from Roche.

For inhibition curves cf. Section SI.5 of the Supplementary Materials.

## 4. Conclusions

We have successfully prepared a disulfide-linked conjugate between a novel epothilone analog and the (purported) EGFR-binding peptide GE11, and we have determined its microtubule-binding affinity and its in vitro antiproliferative activity against EGFR-overexpressing cells and cells being devoid of EGFR. While the conjugate had been designed to specifically target EGFR-overexpressing cells, its in vitro antiproliferative activity was found to be independent of EGFR expression status, at least for the limited number of cell lines evaluated. While our study has been limited in scope, in combination with other data in the literature, our findings could suggest that GE11 does not represent an effective targeting moiety for EGFR-overexpressing tumor cells, and even less so in combination with a disulfide linker (contrary to our original working hypothesis).

Independent of the uncertainties surrounding the activity of the specific epothilone conjugate **4** that we have investigated in this study, our work does demonstrate that the new epothilone analog **1** can serve as highly active template for the construction of targeted prodrugs. The compound exhibits nM antiproliferative activity against a variety of cell lines and offers a readily accessible, sterically unencumbered primary hydroxyl group for chemical manipulation; the requisite partially protected precursor for these manipulations (i.e., intermediate **13**) can be prepared on a scale that allows the production of reasonable amounts of prodrugs for subsequent biochemical, cell biological, and pharmacological studies. The preparation of such prodrugs will be the subject of future studies in our laboratory.

## Figures and Tables

**Table 1:** Binding constants of epothilone analogs **1**, **14**, and **16** and of epothilone-CE11 conjugate **4** for stabilized microtubules ^1^.

Compound	K_b_ [10^6^ M^−1^]
**1**	78.5 ± 17.2
**14**	5.4 ± 0.8
**16**	9.8 ± 0.3
**4**	9.3 ± 0.5
**CysCE11**	0.05 ± 0.02
**Epo A**	31.7 ± 3.1
**Docetaxel**	26.7 ± 3.3

^1^ Association constant K_b_ with glutaraldehyde-stabilized microtubules at 35 °C, determined as described in [42]. Numbers are average values from 3 independent experiments ± standard deviation.

**Table 2:** Antiproliferative activity of epothilone analogs **1** and **16** and of epothilone-CE11 conjugate **4** against human cancer cell lines (IC_50_ [nM]) ^1^.

Compound	A431	SW480	HEK293
**1**	3.32 ± 0.60	17.56 ± 3.50	0.87 ± 0.13
**16**	8.37 ± 0.81	8.91 ± 1.26	2.08 ± 0.53
**4**	74.74 ± 6.65	180.8 ± 25.9	31.09 ± 6.21
**Epo A**	2.94 ± 0.30	14.52 ± 1.09	2.71 ± 0.62

^1^ Cells were incubated with test compounds for 72 h. A431: epidermoid squamous cell carcinoma cells; SW480: colorectal adenocarcinoma cells; HEK293: embryonic kidney cells. Numbers presented are average values from three independent experiments ± standard deviation. For experimental details see the Supplementary Materials.

## Supplementary Materials

Supplementary materials can be found at https://www.mdpi.com/1422-0067/20/5/1113/s1.
