# Supplementary material for: Synthesis, Microtubule-Binding Affinity, and Antiproliferative Activity of New Epothilone Analogs and of an EGFR-Targeted Epothilone-Peptide Conjugate
Source: Int J Mol Sci. 2019 Mar 5;20(5):1113. doi: 10.3390/ijms20051113 (PMC6429585; doi:10.3390/ijms20051113)

Supporting Information

Synthesis, Microtubule-Binding Affinity and Antiproliferative Activity of New Epothilone Analogs and of an EGFR-Targeted Epothilone-Peptide Conjugate

Fabienne Zdenka Gaugaz, Andrea Chicca, Mariano Redondo, Isabel Barasoain, José Fernando Díaz, and Karl-Heinz Altmann*

SI.1. EGFR Expression Quantification

EGFR expression levels were determined by treating cells with 1 μg/ml Cetuximab or human IgG isotype antibody and staining with a secondary Alexa Fluor 647 goat anti-human IgG.[49] The fluorescence was quantified by FACS on a FACSCanto device with a FACSDiva software and later analyzed with FlowJo (Fig. S1). Cetuximab was purchased from Merck, Alexa Fluor 647 goat anti-human IgG, secondary A21445, and human IgG isotype control were from Invitrogen. As MCF-7 cells showed no expression of EGFR, no further experiments were carried out with this cell line.

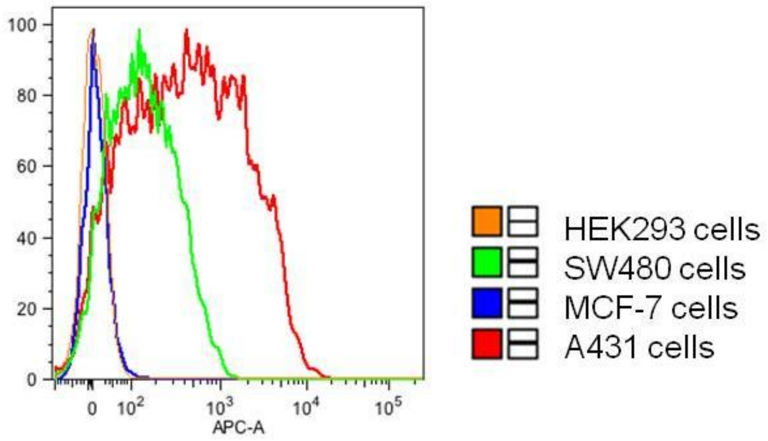

Figure S1: Relative quantification of EGF receptors by FACS.

*SI.2. Stability of conjugate **4** in cell culture medium*

The stability of **4** in cell culture medium was evaluated by analytical RP-HPLC. 5 μL of a 8.6 mM solution of **4** in DMSO were added to 495 μL supplemented cell culture medium and incubated at 37°C, 5% CO_2, so as to mimic the conditions of the cytotoxicity assays. At predetermined time points, 40 μL aliquots were removed from the mixture and treated with one volume of acetonitrile. The mixture was centrifuged and 10 μL of the supernatant were analyzed by analytical HPLC: H_2O with 0.1% TFA (A) / acetonitrile/H_2O 8/2 with 0.05% TFA (B). Linear gradient from 5% B to 80% B over 30 min. TFA = trifluoracetic acid.

SI.3. Disulfide cleavage asssay

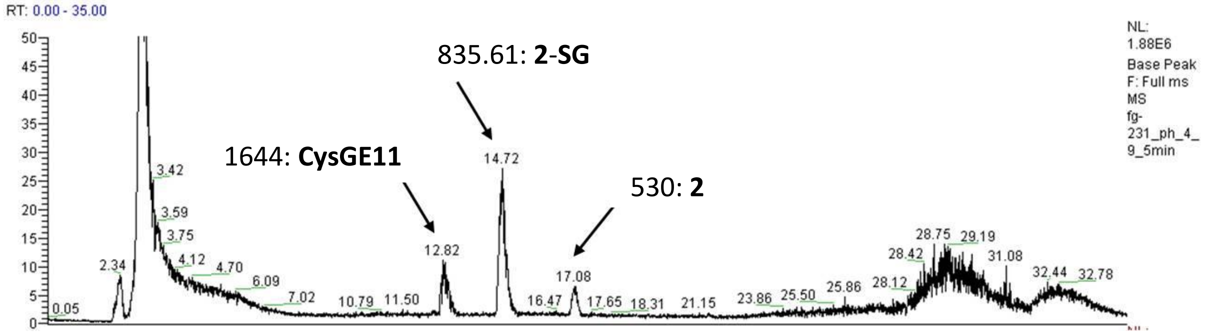

Figure S2. LC/MS trace of the glutathione (GSH) cleavage solution at pH 4.9 after 5 min. For details see "Materials and Methods".

SI.4. Determination of microtubule-binding affinity

Preparation of Stabilized Microtubules

60 mg of frozen tubulin were thawed and loaded on a Sephadex G-25 column pre-equilibrated with cold 1 mM EGTA, 3.4 M glycerol, 10 mM sodium phosphate pH 6.8 buffer supplemented with 0.1 mM GTP. It was eluted with 12 ml buffer. Fractions were collected and analyzed by spectrophotometry at 295 nm on a Thermo Evolution 300LC device. Positive fractions (absorption above 0.5) were centrifuged for 15 min at 50'000 rpm at 4 °C in a TLA 120 rotor in an Optima TLX centrifuge to remove tubulin aggregates. The tubulin concentration of the supernatant was determined by spectrophotometry at a 1:20 dilution of 10 mM sodium phosphate buffer containing 1% SDS. The concentration was calculated with an extinction coefficient of 107'000 $M^{-1}cm^{-1}$ at 276 nm.

The tubulin was polymerized by adding GTP to a final concentration of 1 mM and $MgCl_2$ to 6 mM. It was incubated at 37 °C for 40 min. 4 µL/ml of 50% glutaraldehyde solution were added to crosslink the microtubules. It was incubated for 10 min at 37 °C. The excess glutaraldehyde was quenched by pouring the MTx solution into 60 µL/mL of a freshly prepared 1 M sodium borohydride solution on ice. Foam developed over 10 min on ice. It was collected and shortly spun down (10 sec) by centrifugation. The resulting solution without foam was collected and loaded into a pre-hydrated dialysis cassette (Side-A-Lyzer 0.5-3 ml Thermo Scientific). It was dialyzed for 16 h at 4 °C in glycerol assembly buffer (GAB) consisting of cold 1 mM EGTA, 3.4 M glycerol, 6 mM $MgCl_2$, 10 mM sodium phosphate pH 6.5 buffer supplemented with 0.1 mM GTP. The MTx were recovered and kept on ice. The concentration was measured spectrophotometrically as previously. The concentration was calculated with compensation of the GTP absorption peak at 254 nm with the following extinction coefficients: Abs (276) = x • 107000 + 8800 • y and Abs (254) = x • 64200 + 13400 • y; x being the MTx concentration in [M].

The number of free binding sites was determined with a displacement assay of Flutax-2 by docetaxel measured by fluorometry (Fluoromax-2 device). 500 µl of a solution 2 µM in MTx and 5 µM in Flutax-2 was prepared. 200 µL of this solution were supplemented with docetaxel to a final concentration of 100 µM. This solution and 200 µL of the untreated solution were ultracentrifuged in two vials at 50'000 rpm and rt for 20 min. 100 µL supernatant of both samples were supplemented with 400 µL 1% SDS 10 mM sodium phosphate buffer (20 % GAB). The pellet was resuspended in 200 µL 1% SDS 10 mM sodium phosphate buffer. 100 µL of this solution were supplemented with 300 µL 1% SDS 10 mM sodium phosphate buffer and 100 µL GAB. A calibration curve with Flutax-2 in 80% 1% SDS 10 mM sodium phosphate buffer and 20% GAB with 5 µM, 2 µM, 1 µM, 0.5 µM, 0.2 µM, 0.1 µM and blank was measured together with the samples. Excitation occurred at 495 nm and emission at 520 nm. The calibration curvee was checked for saturation.

The concentration of supernatant and pellet samples was calculated with the calibration curved and multiplied by the dilution factor (5). The obtained concentration of the Flutax-2 and docetaxel was substracted from the Flutax-2 sample concentration for both pellet and supernatant. The free binding site concentration was obtained by multiplying the previously obtained MTx concentration by the obtained pellet factor, while considering the used MTx concentration. Drops of the MTx solution were frozen with liquid nitrogen and stored at - 80 °C.

Determination of the Microtubule Binding Affinity

The equilibrium binding constants of the different compounds were measured by displacement of 50 nM Flutax-2 from the paclitaxel binding site of 50 nM cross-linked stabilized microtubules (MTx, 50 nM paclitaxel binding site, as determined above). Each assay was done in triplicate at least. 96-well plates of MTx and Flutax-2 solution in GAB (glycerol assembly) buffer were treated with serial dilutions of the compounds in DMSO. Controls were untreated wells and MTx and Flutax-2 alone, as well as docetaxel and Epo A serial dilutions. After 40 minutes incubation at rt, the anisotropy was measured at 26, 27, 30, 32, 35, 37, 40 and 42 °C with a POLARSTAR BMG fluorescence polarization microplate reader (for confirmation of irreversible transformations, the temperature order was inversed). Excitation occurred using the 480-P filter and emission with the 520-P filter. Processing of the data was based on previously acquired constants for Flutax-2 for each temperature, as described by Buey [42]. The binding of Flutax-2 in the presence of the compounds (fractional saturation, ν_x) was calculated based on the bound r_o and displaced r_{min} state of Flutax-2 (r_0: no competitor; r_{min}: with 25 μM docetaxel); r_x being the measured anisotropy values and ν_o the binding of Flutax-2 in the absence of competitor:

$$v_x = \frac{v_0 * (r_x - r_{min})}{(r_0 - r_{min})}$$

Binding constants were calculated with the Equigra v5 software based on the best fit of K_a values for Flutax-2 and ν_x assuming unitary stoichiometry of binding. (MT: microtubule, F: Flutax-2, L: ligand, S: sites, b: bound, f: free, t: total) (Fig. S3).

$$\text{MT-F} + \text{L} \rightleftharpoons \text{MT-L} + \text{F} \quad K_a(L) = [L]_b/[S]_f \bullet [L]_f$$

Temperature [°C]	Flutax-2 K_a [10^7 M^{-1}]	ν_o
**26**	6.5	0.578
**27**	5.9	0.563
**30**	4.6	0.522
**32**	4.2	0.508
**35**	3.0	0.451
**37**	2.2	0.398
**40**	2.0	0.382
**42**	1.8	0.364

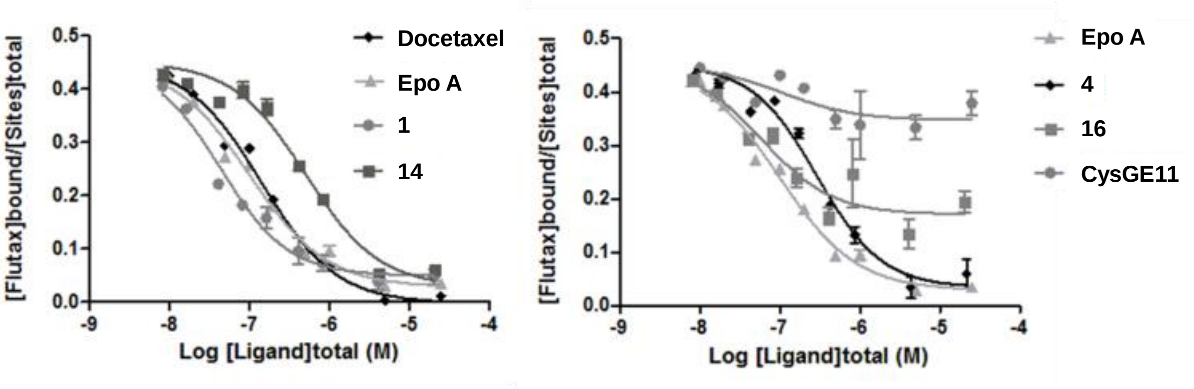

Figure S3. Competition between Flutax-2 and epothilones for the paclitaxel binding site: Displacement of the fluorescent taxoid Flutax-2 (50 nM) from microtubule binding sites (50 nM) by docetaxel, epothilone analogs and CysGE11 at 35°C.

SI.5. Cell growth inhibition curves

For experimental details *cf*. "Materials and Methods".

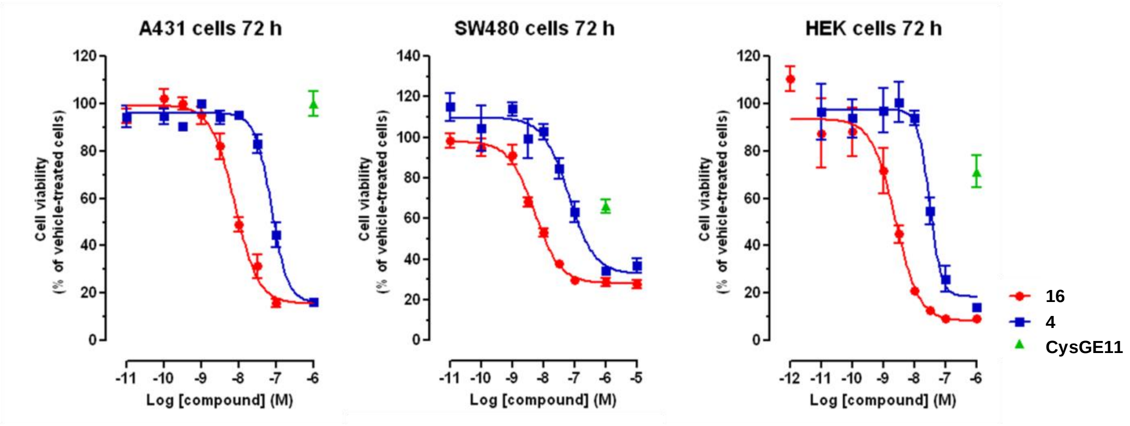

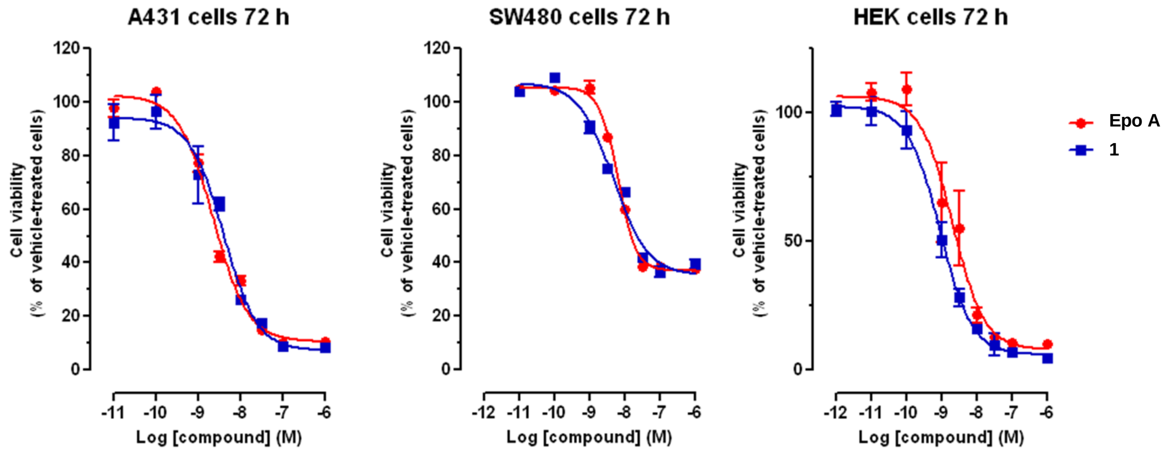

Supplement: Supplementary file 1 [file ijms-20-01113-s001.pdf]
